# Serum neurofilament light and glial fibrillary acidic protein levels are not associated with wearing-off symptoms in natalizumab-treated multiple sclerosis patients

**DOI:** 10.1177/13524585241293940

**Published:** 2024-11-04

**Authors:** Alyssa A Toorop, Mark HJ Wessels, Lynn Boonkamp, Liza MY Gelissen, E Hoitsma, Esther MPE Zeinstra, Luuk C van Rooij, Caspar EP van Munster, Anke Vennegoor, Jop P Mostert, Beatrijs HA Wokke, Nynke F Kalkers, Erwin LJ Hoogervorst, Jeroen JJ van Eijk, Christiaan M Roosendaal, Jolijn J Kragt, Marijke Eurelings, Jessie van Genugten, Jessica Nielsen, LGF Sinnige, Mark E Kloosterziel, Edo PJ Arnoldus, Gert W van Dijk, Willem H Bouvy, Eva MM Strijbis, Bob W van Oosten, Brigit A de Jong, Bernard MJ Uitdehaag, Theo Rispens, Joep Killestein, Zoé LE van Kempen, Charlotte E Teunissen

**Affiliations:** Department of Neurology, MS Center Amsterdam, Amsterdam Neuroscience, Amsterdam UMC, Vrije Universiteit Amsterdam, Amsterdam, The Netherlands; Department of Neurology, MS Center Amsterdam, Amsterdam Neuroscience, Amsterdam UMC, Vrije Universiteit Amsterdam, Amsterdam, The Netherlands; Neurochemistry Laboratory, Department of Laboratory Medicine, Amsterdam University Medical Center, Vrije Universiteit Amsterdam, Amsterdam Neuroscience, Amsterdam, The Netherlands; Department of Neurology, MS Center Amsterdam, Amsterdam Neuroscience, Amsterdam UMC, Vrije Universiteit Amsterdam, Amsterdam, The Netherlands; Department of Neurology, MS Center Alrijne Hospital, Leiden, The Netherlands; Department of Neurology, Isala, Meppel, The Netherlands; Department of Neurology, Maasstad Hospital, Rotterdam, The Netherlands; Department of Neurology, Amphia, Breda, The Netherlands; Department of Neurology, Flevoziekenhuis, Almere, The Netherlands; Department of Neurology, Rijnstate Hospital, Arnhem, The Netherlands; Department of Neurology, Erasmus Medical Center, Rotterdam, The Netherlands; Department of Neurology, OLVG, Amsterdam, The Netherlands; Department of Neurology, St. Antonius Ziekenhuis, Utrecht, The Netherlands; Department of Neurology, Jeroen Bosch Ziekenhuis/Hospital, ‘s-Hertogenbosch, The Netherlands; Department of Neurology, Slingeland Hospital, Doetinchem, The Netherlands; Department of Neurology, Reinier de Graaf Hospital, Delft, The Netherlands; Department of Neurology, Spaarne Gasthuis, Haarlem, The Netherlands; Department of Neurology, Ziekenhuisgroep Twente, Almelo, The Netherlands; Department of Neurology, Ommelander Ziekenhuis, Scheemda, The Netherlands; Department of Neurology, Medisch Centrum Leeuwarden, Leeuwarden, The Netherlands; Department of Neurology, Wilhelmina Hospital, Assen, The Netherlands; Department of Neurology, Elisabeth-TweeSteden Hospital, Tilburg, The Netherlands; Department of Neurology, Canisius Wilhelmina Hospital, Nijmegen, The Netherlands; Department of Neurology, Diakonessenhuis Hospital, Utrecht, The Netherlands; Department of Neurology, MS Center Amsterdam, Amsterdam Neuroscience, Amsterdam UMC, Vrije Universiteit Amsterdam, Amsterdam, The Netherlands; Department of Neurology, MS Center Amsterdam, Amsterdam Neuroscience, Amsterdam UMC, Vrije Universiteit Amsterdam, Amsterdam, The Netherlands; Department of Neurology, MS Center Amsterdam, Amsterdam Neuroscience, Amsterdam UMC, Vrije Universiteit Amsterdam, Amsterdam, The Netherlands; Department of Neurology, MS Center Amsterdam, Amsterdam Neuroscience, Amsterdam UMC, Vrije Universiteit Amsterdam, Amsterdam, The Netherlands; Sanquin Diagnostic Services, Amsterdam, the Netherlands; Department of Immunopathology, Sanquin Research and Landsteiner Laboratory, Amsterdam UMC, University of Amsterdam, Amsterdam, The Netherlands; Department of Neurology, MS Center Amsterdam, Amsterdam Neuroscience, Amsterdam UMC, Vrije Universiteit Amsterdam, Amsterdam, The Netherlands; Department of Neurology, MS Center Amsterdam, Amsterdam Neuroscience, Amsterdam UMC, Vrije Universiteit Amsterdam, Amsterdam, The Netherlands; Neurochemistry Laboratory, Department of Laboratory Medicine, Amsterdam University Medical Center, Vrije Universiteit Amsterdam, Amsterdam Neuroscience, Amsterdam, The Netherlands

**Keywords:** Multiple sclerosis, biomarkers, natalizumab, treatment response

## Abstract

**Background:**

Biomarkers of neuronal and axonal damage (serum neurofilament light (sNfL) and serum glial fibrillary acidic protein (sGFAP)) may provide insight into the aetiology of natalizumab wearing-off symptoms (WoSs).

**Objectives:**

We investigated the longitudinal association between and predictive value of sNfL and sGFAP and the occurrence of WoS in MS patients treated with natalizumab.

**Methods:**

We performed longitudinal measurements of sNfL and sGFAP in NEXT-MS trial participants who completed a questionnaire about WoS.

**Results:**

A total of 364 participants were included. In total, 55.5% presented with WoS and 44.5% without WoS during natalizumab treatment. Longitudinal analyses showed no association between sNfL and sGFAP levels and WoS at any timepoint. Biomarker levels at baseline did not predict first-time WoS occurrence.

**Conclusion:**

Acute and chronic neuronal and axonal damage are most likely not the underlying cause of WoS.

## Introduction

Wearing-off symptoms (WoSs) are reported by a significant part of multiple sclerosis (MS) patients during treatment with natalizumab.^1^ WoS are MS-related symptoms, such as fatigue, that increase towards the next natalizumab dosing and usually disappear shortly afterwards. So far, there is no evidence that MS disease activity is associated with WoS.^2^ Only higher body mass index (BMI) and body weight were reported to be associated with WoS.^1,3^

Blood biomarkers of neuronal and axonal damage such as serum neurofilament light (sNfL) and serum glial fibrillary acidic protein (sGFAP) are increasingly used to monitor disease activity and treatment response.^4,5^ Biomarkers can also be indicative of smouldering disease, as elevated levels of sNfL are associated with chronic white matter inflammation and microglial activation.^6^ The sGFAP levels have been associated with disability, lesion load and neurodegeneration.^7^ Measurement of in vivo biomarkers might provide insight into the biological underpinnings of WoS.

The aim of our study was to investigate the association between sNfL and sGFAP and the occurrence of WoS in MS patients treated with natalizumab. In addition, we studied whether these biomarkers can predict the occurrence of WoS during further treatment.

## Methods

### Study design and participants

Patients with relapsing-remitting MS who received at least six natalizumab infusions were included in the NEXT-MS trial between February 2020 and April 2022.8 The NEXT-MS trial was an investigator-initiated nonrandomized multicenter study on personalized extended dosing based on natalizumab serum concentrations. Participants who completed at least one WoS questionnaire were included in this substudy.

### Study procedures and outcomes

Participants received questionnaires about WoS at three time points: start of the NEXT-MS trial, year 1 and year 2. Questions regarding WoS were similar to previous studies on WoS in our centre and captured ever experiencing WoS since natalizumab initiation (baseline) or in the past year (years 1 and 2).^1^ Patients were counted as experiencing WoS if they answered these questions positively. Serum was collected after screening and during the study at natalizumab infusions and grouped based on weeks from baseline. Left-over serum after measuring natalizumab concentrations (mean 2 samples per patient, mean time between samples 10.3 months) was used to measure biomarker levels in one batch (Simoa^®^ Neurology 4-Plex E Advantage Kit, Quanterix, Billerica, MA, USA).^4^ Biomarker results were expressed as levels and delta values.

### Statistical analyses

Patients were divided between those experiencing WoS during treatment with natalizumab and those that did not. Clinical characteristics were compared between groups using chi-square test, *t*-test and Mann–Whitney *U*-test. Further analysis into frequency (never WoS vs. often/always) was performed afterwards. The association between longitudinal biomarker levels and WoS was investigated using Generalized Estimating Equations (GEEs).

Next, the predictive values of biomarker levels at baseline for first-time WoS converters (patients experiencing WoS for the first time after baseline) were tested using logistic regression. Corrections for sex, age, BMI and Expanded Disability Status Scale (EDSS) were performed in GEE and logistic regression. A *p*-value < 0.05 was considered significant. Statistical analyses and visualizations were performed with R software version 4.3.2.

## Results

### Participants

A total of 364 participants started the NEXT-MS trial and completed at least one WoS questionnaire (mean = 2). We identified 202 (55.5%) participants with WoS and 162 (44.5%) participants without WoS during natalizumab treatment. Thirty-three patients experienced WoS for the first time during follow-up. EDSS at baseline was higher in participants with WoS (Table 1).

**Table 1.** Baseline characteristics of included participants.

	Never experienced WoS	Ever experienced WoS	Total	*p*
	(*N* = 162)	(*N* = 202)	(*N* = 364)	
Sex, *n* (%)				
Male	33 (20.4%)	43 (21.3%)	76 (20.9%)	.93
Female	129 (79.6%)	159 (78.7%)	288 (79.1%)	
Age, years (IQR)	39.0 (33.0–49.0)	40.0 (33.0–50.0)	40.00 (33.0–50.0)	.91
BMI at baseline, kg/m^2^ (IQR)	24.44 (21.95–27.70)	24.86 (21.50–28.18)	24.69 (21.60–28.04)	.91
EDSS, (IQR)	2.5 (1.5–4.0)	3.0 ( 2.0–4.4)	3.0 (2.0–4.0)	.0022
JCV-status, *n* (%)				
Negative	138 (85.2%)	169 (83.7%)	307 (84.3%)	.8
Positive	24 (14.8%)	33 (16.3%)	57 (15.7%)	
New/newly enlarged T2 lesions on MRI^a^, *n* (%)				
No activity	145 (89.5%)	181 (89.6%)	326 (89.6%)	1
Activity	17 (10.5%)	21 (10.4%)	38 (10.4%)	
Extended dosing group, *n* (%)				
SID group	18 (11.1%)	39 (19.3%)	57 (15.7%)	.098
EID10 group	113 (69.8%)	130 (64.4%)	243 (66.8%)	
EID5 group	31 (19.1%)	33 (16.3%)	64 (17.6%)	
Duration NTZ treatment, years (IQR)	4.22 (1.31–8.71)	3.98 (1.67–7.50)	4.05 (1.50–8.20)	.85
NfL at baseline, pg/mL (IQR)	9.51 (7.17–13.02)	10.22 (7.40–13.16)	9.81 (7.22–13.1)	.5
GFAP at baseline, pg/mL (IQR)	66.86 (50.95–90.83)	70.75 (53.41–94.36)	68.03 (51.70–92.71)	.71

WoS: wearing-off symptom; BMI: body mass index; JCV: John–Cunningham virus; sNfL: serum neurofilament light; sGFAP: serum glial fibrillary acidic protein; EDSS: Expanded Disability Status Scale; NTZ: natalizumab; SID: standard interval dosing (treatment every 4 weeks); EID10: extended interval dosing with an aim drug trough concentration of 10 µg/mL; EID5: extended interval dosing with an aim drug trough concentration of 5 µg/mL.^8^

Values are depicted as medians with interquartile ranges or frequencies with percentages. Clinical characteristics were compared between groups using the chi-square test for categorical variables, the *t*-test for normally distributed continuous variables and the Mann–Whitney *U* test for non-normally distributed continuous variables.

a Baseline MRI scan of the NEXT-MS trial.

### Biomarkers and WoSs

**Figure 1.**
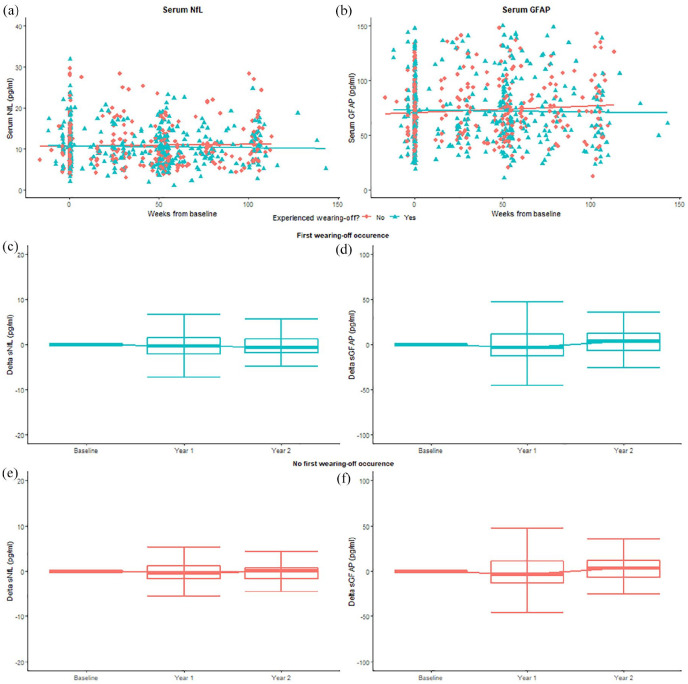
sNfL and sGFAP levels over time. (a) and (b) The x-axis displays continuous time points (weeks from baseline). The y-axis displays sNfL levels in pg/mL (a) and sGFAP levels in pg/mL (b). Baseline represents the start of extended interval dosing for each dose. Samples for the current study were retrieved at the start of the study, year 1 and last follow-up. The figures illustrate no significant difference in sNfL and sGFAP levels over time between participants with WoS and without WoS. (c)–(f) The x-axis displays timepoints (baseline, year 1 and year 2). The y-axis displays the absolute difference in sNfL ((c) and (e)) and sGFAP ((d) and (f)) levels compared to baseline. The figures illustrate no significant change in biomarker levels between participants with first-time WoS occurrence during follow-up and those without. WoS: wearing-off symptom; sNfL: serum neurofilament light; sGFAP: serum glial fibrillary acidic protein.

Figure 1 shows the associations between biomarkers and WoS over time. GEE analyses showed no significant associations between sNfL (odds ratio [OR] = 1.00, 95% confidence interval [CI] = [1.00–1.00], *p* = 0.83) or sGFAP (OR = 1.00, 95% CI = [1.00–1.00], *p* = 0.45) at any timepoint and the occurrence of WoS. There were no changes after correction for confounders (sNfL: OR = 1.0, 95% CI = [1.00–1.00], *p* = 0.79; sGFAP: OR = 1.00, 95% CI = [1.00–1.00], *p* = 0.35). This remained insignificant when comparing never versus often/always WoS.

Furthermore, GEE analyses showed no significant associations between percentual changes in biomarker levels and first-time WoS occurrence. This was observed for both percentual changes in follow-up levels compared to baseline (sNfL: OR = 1.0, 95% CI = [1.00–1.00], *p* = 0.39; sGFAP: OR = 1.00, 95% CI = [1.00–1.00], *p* = 0.13), as well in percentual changes between consecutive measurements (sNfL: OR = 1.0, 95% CI = [1.00–1.00], *p* = 0.99; sGFAP: OR = 1.00, 95% CI = [1.00–1.00], *p* = 0.10). This remained after correction for confounders.

Logistic regression analyses showed that sNfL at baseline (OR = 1.02, 95% CI = [0.95–1.06], *p* = 0.70) and sGFAP at baseline (OR = 1.00, 95% CI = [1.00–1.00], *p* = 0.98) did not predict first-time WoS occurrence. These results did not change after correction for confounders (sNfL: OR = 1.02, 95% CI = [0.96–1.08], *p* = 0.37; sGFAP: OR = 1.00, 95% CI = [0.99–1.01], *p* = 0.85).

## Discussion

The results of our study could not determine neuronal and axonal damage as an underlying cause for WoS, as we found no evidence that WoS are associated with sNfL and sGFAP levels at any timepoint.

We observed a higher EDSS score in participants with WoS at baseline but no significantly higher BMI. Both findings were not consistently replicated by other studies.^1,2,9^ Smouldering disease activity is gaining more attention with regard to disease progression independent of relapses, possibly causing MS-related symptoms and disability without radiological activity . Elevated sNfL was associated with chronic white matter inflammation and microglial activation previously, while sGFAP has been associated with higher lesion loads and EDSS scores.^6,7^ It is reassuring that WoS are likely not reflecting reoccurring neuronal and axonal damage, confirming previous findings in a smaller cohort indicating no elevation of sNfL with WoS.^2^

Former studies showed no association with WoS and MS activity.^1,2^ Other factors such as low natalizumab trough concentrations or presence of serum cytokines were also not associated with WoS.^1,9^ There was no difference in WoS prevalence between participants with standard- or extended dosing in our study.^10^ WoS therefore might reflect a nocebo effect.^1^

Limitations of this study include the nonrandomized study design. WoSs were self-reported and remain subjective due to recall and attribution bias. Strengths include evaluation of prospectively collected longitudinal data with measurements of biomarkers in relation to WoS.

In conclusion, longitudinal analyses showed sNfL and sGFAP levels were not associated with natalizumab WoS, indicating that acute and chronic neuronal and axonal damage are most likely not the underlying cause.
